# On multi-strain model for Hepatitis C

**DOI:** 10.1186/1753-4631-5-6

**Published:** 2011-08-03

**Authors:** E Ahmed, HA El-Saka

**Affiliations:** 1 Mathematics Department, Faculty of Science, Mansoura University, 35516, Mansoura, Egypt; 2 Mathematics Department, Damietta Faculty of Science, Mansoura University, 34517, New Damietta, Egypt

## Abstract

In this paper we present a multi-strain model for hepatitis C virus (HCV) including an immune response term. The model is presented and discussed. Also we argue that the added multi-strain term represents some basic properties of the immune system and that it should be included to study longer term behavior of the disease.

## 1. Introduction

Hepatitis C is one of many serious infectious diseases affecting humans. An estimated about 170 million people worldwide are infected with hepatitis C. No vaccine against hepatitis C is available. Egypt has the highest rate of infections by hepatitis C virus (HCV) genotype 4. Chronic HCV infection is the main cause leading to liver transplant or death [1]. Antiviral therapy has been used to treat chronically HCV infected patients. Relatively successful treatment contains pegylated interferon (PEG-IFN) and ribavirin (RBV). Typical response ate biphasic beginning with rapid viral decline phase followed by slower decline phase. Sometimes there is more complicated behavior but they will not be discussed here. Extensive and impressive studies have been done by Perelson and his group [4-6]. Here we present a multi-strain generalization of a basic HCV model [4] including the immune response (Ir) term proposed in [3].

Why do we need mathematical models for HCV?

i) The ideal research group should contain both medical doctors (MD) and mathematicians/and or biophysicists c.f. Perelson group (Santa Fe).

ii) A standard result is that multi-drugs have to be used for treatment. This does not seem to be the case for hepatitis C. Only Peg-IFN is used. Ribavirin (RBV) cannot be considered as independent drug since, if used independently, it has no effect on HCV.

iii) Multi-strain effects are relevant to HCV. Mathematical models for multi-strain viruses can be quite useful. In fact trying to anticipate the new strains experimentally is quite difficult [7].

iv) Spatial effects of epidemics can be important. Typically they are difficult to quantify. Mathematical models can help in this problem.

In sec.2 the model is presented and discussed. Also we argue that the added immune response term represents some basic properties of the immune system and that it should be included to study longer term behavior of the disease.

## 2. The model and conclusions

The fractional order hepatitis C virus (HCV) model is given by

(1) $$\begin{array}{c} D^{\alpha }T=s-d\;T-\left(1-\eta \right)\beta \;V\;T,\\ D^{\alpha }I=\left(1-\eta \right)\beta \;V\;T-\delta \;I\left(1-I∕c_{2}\right),\\ D^{\alpha }V=\left(1-\varepsilon_{p}\right)p\;I-c\;V, \end{array}$$

Where 0 <*α *≤ 1, *T *represents uninfected hepatocytes, *I *represents infected hepatocytes and *V *represents virus. The model assumes that uninfected hepatocytes are produced at a constant rate *s*, die at rate, *d*, per cell and are infected at constant rate *β*. Infected hepatocytes are lost at a rate *δ *per cell. Viral particles (virions) are produced at rate *p *per infected hepatocyte and cleared at rate *c *per virion. Chronic HCV infection is treated using interferon-a in combination with the antiviral drug ribavirin. Interferon-a acts primarily by blocking the production/release of new virus, although we also allow for a treatment effect in blocking *de novo *infection. The efficacy of treatment in blocking virion production and reducing new infections are described by two parameters, *ε_p _*and *η*, respectively. For example, a treatment efficacy in blocking virion production of 95% corresponds to *ε_p _= *0.95.

Setting V to be proportional to I one gets the reduced system:

(2) $$\begin{array}{lll} \text{DT} & =\text{s - d T - a}\;\text{I}\;\text{T, }\; & \text{(1)}\\ \text{DI} & =\text{a}\;\text{I}\;\text{T - b}\;\text{I(1 - }\frac{\text{I}}{\text{c}}\text{)}\text{.}\; & \text{(2)}\\ & \; & \text{(3)} \end{array}$$

Where *a*, *b*, *c*, *d*, *s *are positive constants. There are three equilibrium points of the system (2). The first is the disease free $\text{(T}=\frac{\text{s}}{\text{d}}\text{, I}=\text{0)}$ and two endemic states (T_1_, I_1_) and (T_2_, I_2_) where

(3) $$\begin{array}{c} {\text{T}}_{\text{j}}=\text{b}(1-\frac{{\text{I}}_{\text{j}}}{\text{c}})/\text{a},\;\;\;\;\;\;\;\text{j}=\text{1,2,}\\ {\text{I}}_{\text{1}}=\frac{c}{2}\text{\{ (1 - }\frac{\text{d}}{\text{ac}}\text{)}+{\left[{\left(1-\frac{\text{d}}{\text{ac}}\right)}^{\text{2}}\text{ - (}\frac{\text{4}}{\text{c}}\text{)(}\frac{\text{s}}{\text{b}}\text{ - }\frac{\text{d}}{\text{a}})\right]}^{\text{(1/2)}}\text{\}},\\ {\text{I}}_{\text{2}}=\frac{c}{2}\text{\{ (1 - }\frac{\text{d}}{\text{ac}}\text{)}-{[{(1-\frac{\text{d}}{\text{ac}}\text{)}}^{\text{2}}\text{ - (}\frac{\text{4}}{\text{c}}\text{)(}\frac{\text{s}}{\text{b}}\text{ - }\frac{\text{d}}{\text{a}}\text{)]}}^{\text{(1/2)}}\text{\}}. \end{array}$$

The locally asymptotic stability condition for the disease free state is

(4) $$\frac{s}{d}<\frac{b}{a}.$$

The locally asymptotic stability conditions for the two endemic states are:

(5) $$\text{0}<{\text{I}}_{\text{j}}<\frac{\text{d}}{\text{(b/c - a)}},$$

(6) $$\text{2(}\frac{\text{as}}{\text{b}}\text{ - d)}>\text{(a - }\frac{\text{d}}{\text{c}}\text{)}{\text{I}}_{\text{j}}\text{, j}=\text{1,2}\text{.}$$

## 3. The multi-strain HC model

The model is given by:

(7) $$\begin{array}{c} \text{DT}=\text{s - d}\;\text{T - }{\text{a}}_{\text{1}}{\text{I}}_{\text{1}}\text{T - }{\text{a}}_{\text{2}}{\text{I}}_{\text{2}}\text{T,}\\ \text{D}{\text{I}}_{\text{1}}={\text{a}}_{\text{1}}{\text{I}}_{\text{1}}\text{T - }{\text{b}}_{\text{1}}{\text{I}}_{\text{1}}\text{(1 - }{\text{I}}_{\text{1}}\text{/}{\text{c}}_{\text{1}}\text{)}+{\text{g}}_{\text{1}}{\text{I}}_{\text{2}}\;\text{.}\\ \text{D}{\text{I}}_{\text{2}}={\text{a}}_{\text{2}}{\text{I}}_{\text{2}}\text{T - }{\text{b}}_{\text{2}}{\text{I}}_{\text{2}}\text{(1 - }{\text{I}}_{\text{2}}\text{/}{\text{c}}_{\text{2}}\text{)}+{\text{g}}_{\text{2}}{\text{I}}_{\text{1}}\text{,} \end{array}$$

where *a*_1_, *a*_2_, *b*_1_, *b*_2_, *c*_1_, *c*_2_, *d*, *s*, *g*_1_, *g*_2 _are positive constants.

There are several equilibrium solutions: The disease free state (T = *s*/*d*, I_1 _= I_2 _= 0). Four one strain endemic states given by (T_1j_, I_1j_, 0) and (T_2j_, 0, I_2j_), j = 1, 2 and:

(8) $$\begin{array}{c} {\text{T}}_{\text{1j}}=\text{(}{\text{b}}_{\text{1}}\text{/}{\text{a}}_{\text{1}}\text{)(1 - }{\text{I}}_{\text{1j}}\text{/}{\text{c}}_{\text{1}}\text{),}\\ {\text{I}}_{\text{11}}=\text{\{ (1 - d/}{\text{a}}_{\text{1}}{\text{c}}_{\text{1}}\text{)}+{\text{[}{\text{(1 - d/}{\text{a}}_{\text{1}}{\text{c}}_{\text{1}}\text{)}}^{\text{2}}\text{ - (4/}{\text{c}}_{\text{1}}\text{)(s/}{\text{b}}_{\text{1}}\text{ - d/}{\text{a}}_{\text{1}}\text{)]}}^{\text{(1/2)}}\text{\}}{\text{c}}_{\text{1}}\text{/2,}\\ {\text{I}}_{\text{12}}=\text{\{ (1 - d/}{\text{a}}_{\text{1}}{\text{c}}_{\text{1}}\text{) - }{\text{[}{\text{(1 - d/}{\text{a}}_{\text{1}}{\text{c}}_{\text{1}}\text{)}}^{\text{2}}\text{ - (4/}{\text{c}}_{\text{1}}\text{)(s/}{\text{b}}_{\text{1}}\text{ - d/}{\text{a}}_{\text{1}}\text{)}\text{]}}^{\text{(1/2)}}\text{\}}{\text{c}}_{\text{1}}\text{/2,}\\ {\text{T}}_{\text{2j}}=\text{(}{\text{b}}_{\text{2}}\text{/}{\text{a}}_{\text{2}}\text{)(1 - }{\text{I}}_{\text{2j}}\text{/}{\text{c}}_{\text{2}}\text{), } \end{array}$$

(9) $$\begin{array}{c} {\text{I}}_{\text{21}}=\text{\{ (1 - d/}{\text{a}}_{\text{2}}{\text{c}}_{\text{2}}\text{)}+{\text{[}{\text{(1 - d/}{\text{a}}_{\text{2}}{\text{c}}_{\text{2}}\text{)}}^{\text{2}}\text{ - (4/}{\text{c}}_{\text{2}}\text{)(s/}{\text{b}}_{\text{2}}\text{ - d/}{\text{a}}_{\text{2}}\text{)]}}^{\text{(1/2)}}\text{\}}{\text{c}}_{\text{2}}\text{/2,}\\ {\text{I}}_{\text{22}}=\text{\{ (1 - d/}{\text{a}}_{\text{2}}{\text{c}}_{\text{2}}\text{) - }{\text{[}{\text{(1 - d/}{\text{a}}_{\text{2}}{\text{c}}_{\text{2}}\text{)}}^{\text{2}}\text{ - (4/}{\text{c}}_{\text{2}}\text{)(s/}{\text{b}}_{\text{2}}\text{ - d/}{\text{a}}_{\text{2}}\text{)}\text{]}}^{\text{(1/2)}}\text{\}}{\text{c}}_{\text{2}}\text{/2}\text{.} \end{array}$$

The multi-strain equilibrium solutions are given by the system:

(10) $$\begin{array}{c} \text{s - dT - }{\text{a}}_{\text{1}}{\text{I}}_{\text{1}}\text{T - }{\text{a}}_{\text{2}}{\text{I}}_{\text{2}}\text{T}=\text{0,}\\ {\text{a}}_{\text{1}}{\text{I}}_{\text{1}}\text{T - }{\text{b}}_{\text{1}}{\text{I}}_{\text{1}}\text{(1 - }{\text{I}}_{\text{1}}\text{/}{\text{c}}_{\text{1}}\text{)}+{\text{g}}_{\text{1}}{\text{I}}_{\text{2}}=\text{0,}\\ {\text{a}}_{\text{2}}{\text{I}}_{\text{2}}\text{T - }{\text{b}}_{\text{2}}{\text{I}}_{\text{2}}\text{(1 - }{\text{I}}_{\text{2}}\text{/}{\text{c}}_{\text{2}}\text{)}+{\text{g}}_{\text{2}}{\text{I}}_{\text{1}}=\text{0}\text{.} \end{array}$$

The local asymptotic stability of the single healthy state is given by

(11) $$\frac{s}{d}<\frac{b_{1}}{a_{1}}\text{, }\;\;\;\;\;\;\frac{s}{d}<\frac{b_{2}}{a_{2}},$$

(12) $$\left(b_{1}-\frac{a_{1}s}{d}\right)\left(b_{2}-\frac{a_{2}s}{d}\right)>g_{1}g_{2}>0.$$

The local asymptotic stability of the one strain endemic states are:

(13) $${\text{h}}_{\text{1}}>\text{0,}\;\;\;\;\;\;\;{\text{h}}_{\text{3}}>\text{0, }\;\;\;\;\;\;\;{\text{h}}_{\text{1}}{\text{h}}_{\text{2}}>{\text{h}}_{\text{3}}.$$

For the state (T_1j_, I_1j_, 0) we have:

(14) $${\text{h}}_{\text{1}}=\text{d}+{\text{b}}_{\text{1}}+{\text{b}}_{\text{2}}+\text{(}{\text{a}}_{\text{1}}\text{ - 2}{\text{b}}_{\text{1}}\text{/}{\text{c}}_{\text{1}}\text{)}{\text{I}}_{\text{1j}}\text{ - (}{\text{a}}_{\text{1}}+{\text{a}}_{\text{2}}\text{)}{\text{T}}_{\text{1j}},$$

(15) $$\begin{array}{c} {\text{h}}_{\text{2}}=-{\text{a}}_{1}\text{d}{\text{T}}_{\text{1j}}\text{ - }{\text{g}}_{\text{1}}{\text{g}}_{\text{2}}+{\text{b}}_{\text{1}}\text{(1 - 2}{\text{I}}_{\text{1j}}\text{/}{\text{c}}_{\text{1}}\text{)(d}+{\text{a}}_{\text{1}}{\text{I}}_{\text{1j}}\text{)}+\\ \text{( - }{\text{a}}_{\text{2}}{\text{T}}_{\text{1j}}+{\text{b}}_{\text{2}}\text{)[d - }{\text{a}}_{\text{1}}{\text{T}}_{1j}+{\text{b}}_{1}+\text{(}{\text{a}}_{\text{1}}\text{ - 2}{\text{b}}_{\text{1}}\text{/}{\text{c}}_{\text{1}}\text{)}{\text{I}}_{\text{1j}}\text{]}, \end{array}$$

and

(16) $$\begin{array}{c} {\text{h}}_{\text{3}}=\text{( - }{\text{a}}_{\text{2}}{\text{T}}_{\text{1j}}+{\text{b}}_{\text{2}}\text{)\{ - }{\text{a}}_{\text{1}}\text{d}{\text{T}}_{\text{1j}}+{\text{b}}_{\text{1}}\text{(1 - 2}{\text{I}}_{\text{1j}}\text{/}{\text{c}}_{\text{1}}\text{)(d}+{\text{a}}_{\text{1}}{\text{I}}_{\text{1j}}\text{)\}}+\\ {\text{g}}_{\text{2}}\text{[ - }{\text{g}}_{\text{1}}\text{(d}+{\text{a}}_{\text{1}}{\text{I}}_{\text{1j}}\text{)}+{\text{a}}_{\text{1}}{\text{a}}_{\text{2}}{\text{I}}_{\text{1j}}{\text{T}}_{\text{1j}}\text{]}\text{.} \end{array}$$

For the state (T_2j_, 0, I_2j_) we have:

(17) $${\text{h}}_{\text{1}}=\text{d}+{\text{b}}_{\text{1}}+{\text{b}}_{\text{2}}+\text{(}{\text{a}}_{\text{2}}\text{ - 2}{\text{b}}_{\text{2}}\text{/}{\text{c}}_{\text{2}}\text{)}{\text{I}}_{\text{2j}}\text{ - (}{\text{a}}_{\text{1}}+{\text{a}}_{\text{2}}\text{)}{\text{T}}_{\text{2j}},$$

(18) $$\begin{array}{c} {\text{h}}_{\text{2}}=-{\text{a}}_{2}\text{d}{\text{T}}_{\text{2j}}\text{ - }{\text{g}}_{\text{1}}{\text{g}}_{\text{2}}+{\text{b}}_{\text{2}}\text{(1 - 2}{\text{I}}_{\text{2j}}\text{/}{\text{c}}_{\text{2}}\text{)(d}+{\text{a}}_{\text{2}}{\text{I}}_{\text{2j}}\text{)}+\\ \text{( - }{\text{a}}_{\text{1}}{\text{T}}_{\text{2j}}+{\text{b}}_{\text{1}}\text{)[d - }{\text{a}}_{\text{2}}{\text{T}}_{2j}+{\text{b}}_{2}+\text{(}{\text{a}}_{\text{2}}\text{ - 2}{\text{b}}_{\text{2}}\text{/}{\text{c}}_{\text{2}}\text{)}{\text{I}}_{\text{2j}}\text{]}, \end{array}$$

and

(19) $$\begin{array}{c} {\text{h}}_{\text{3}}=\text{( - }{\text{a}}_{\text{1}}{\text{T}}_{\text{2j}}+{\text{b}}_{\text{1}}\text{)\{ - }{\text{a}}_{\text{2}}\text{d}{\text{T}}_{\text{2j}}+{\text{b}}_{\text{2}}\text{(1 - 2}{\text{I}}_{\text{2j}}\text{/}{\text{c}}_{\text{2}}\text{)(d}+{\text{a}}_{\text{2}}{\text{I}}_{\text{2j}}\text{)\}}+\\ {\text{g}}_{\text{1}}\text{[ - }{\text{g}}_{\text{2}}\text{(d}+{\text{a}}_{\text{2}}{\text{I}}_{\text{2j}}\text{)}+{\text{a}}_{\text{1}}{\text{a}}_{\text{2}}{\text{I}}_{\text{2j}}{\text{T}}_{\text{2j}}\text{]}\text{.} \end{array}$$

We have the following conclusions:

i) Mathematical models, when done by a group of mathematicians and HCV specialists give relevant results [5].

ii) The term added in equation (1) represents the well known high and low zone tolerance of the immune response (There is no Ir if the antigen number is either too high or too low [2]) since it vanishes for both limits *I *→ 0 and I → *c*_2_. Immune response is significant for long time virus dynamics.

iii) Since multi-drugs are strongly recommended to avoid drug resistance it is proposed that a further drug should be used in addition to Peg-IFN and RBV. Notice that RBV may not be considered as independent drug since, when used alone, it has no effect on HCV.

iv Multi-strain phenomena is quite important. It exists in many diseases e.g. Flu, HCV Tb etc... It may cause the failure of treatment and/or vaccination protocols.

## Competing interests

The authors declare that they have no competing interests.

## Authors' contributions

The two authors contributed equally to this article. All authors read and approved the final manuscript.
